# Sensitivity and specificity of secondary dose calculation for head and neck treatment plans

**DOI:** 10.1002/acm2.14139

**Published:** 2023-09-10

**Authors:** Sonja Wegener, Ruaa Abu Rashed, Otto A. Sauer, Gary Razinskas

**Affiliations:** ^1^ University Hospital Wurzburg Department of Radiation Oncology Wuerzburg Germany; ^2^ Ernst‐Abbe‐Hochschule Jena Jena Germany

**Keywords:** plan‐specific quality assurance, ROC analysis, secondary dose calculation

## Abstract

**Purpose:**

Secondary dose calculation (SDC) with an independent algorithm is one option to perform plan‐specific quality assurance (QA). While measurement‐based QA can potentially detect errors in plan delivery, the dose values are typically only compared to calculations on homogeneous phantom geometries instead of patient CT data. We analyzed the sensitivity and specificity of an SDC software by purposely introducing different errors and determined thresholds for optimal decisions.

**Methods:**

Thirty head and neck VMAT plans and 30 modifications of those plans, including errors related to density and beam modelling, were recalculated using RadCalc with a Monte Carlo algorithm. Decision thresholds were obtained by receiver operating characteristics (ROC) analysis. For comparison, measurement‐based QA using the ArcCHECK phantom was carried out and evaluated in the same way.

**Results:**

Despite optimized decision thresholds, none of the systems was able to reliably detect all errors. ArcCHECK analysis using a 2%/2 mm criterion with a threshold of 91.1% had an area under the curve (AUC) of 0.80. Evaluating differences in recalculated and planned DVH parameter of the target structures in RadCalc with a 2% threshold an AUC of 0.86 was achieved. Out‐of‐field deviations could be attributed to weaknesses in the beam model.

**Conclusions:**

Secondary dose calculation with RadCalc is an alternative to established measurement‐based phantom QA. Different tools catch different errors; therefore, a combination of approaches should be preferred.

## 1 INTRODUCTION

Each intensity‐modulated radiotherapy (IMRT) or volumetric modulated radiotherapy (VMAT) treatment plan is typically checked before patient treatment to verify correct dose calculation, data transfer and application on the linear accelerator. Measurement based as well as calculation‐based approaches exist for the purpose of quality assurance (QA).^1^

Measurement‐based approaches range from point dose measurements to three‐dimensional dose measurements utilizing all sorts of detectors, including ion chambers, diodes, the electronic portal imager (EPID), and gel.^1^, ^2^, ^3^, ^4^, ^5^, ^6^ Typically, either fluences or dose distributions are compared. Comparisons between measured point doses and calculated dose distributions are typically performed by means of gamma analysis. However, gamma analysis has been criticized and dose‐volume‐histogram (DVH) parameters should be preferred to predict clinically relevant errors.^7^ The main drawbacks of measurement‐based QA include the uncertainties in measurement setup and the need for time on the machine.

A number of in‐house or commercial software solutions exist to independently recalculate treatment plans.^8^, ^9^, ^10^, ^11^, ^12^ An overview of products and guidance on commissioning and utilizing a secondary monitor unit (MU) check program was given in a recent task group report.^13^ While the report focuses primarily on point‐dose/MU checks, programs now typically allow for a three‐dimensional dose calculation on the patient anatomy and a comparison of dose maps or assessment of DVH parameters.^14^, ^15^

Decision thresholds are important for users to identify whether a plan is acceptable for irradiation or whether the plan will fail to lead to the desired clinical outcome. The criteria to apply, depend on the QA tool, evaluation methodology and workflow details, and are usually derived individually by institutions.^1^ Receiver‐operating characteristics (ROC) analysis was introduced by Carlone et al. as one systematic way to obtain those thresholds for IMRT plan‐specific QA.^16^ It was since then applied to different QA systems.^3^, ^5^, ^15^, ^17^ Unlike other methods, ROC analysis is performed on a dataset of acceptable and unacceptable plans and derives decision thresholds by maximizing the sensitivity and specificity of a decision criterion.

The performance of earlier versions of the secondary dose calculation program RadCalc has been investigated for various types of treatment machines.^18^, ^19^, ^20^ RadCalc now offers dose calculation on the patient geometry using a Monte Carlo algorithm, which, to the best of our knowledge, has not yet been thoroughly investigated. In this work, ROC analysis was applied to RadCalc's secondary dose calculation QA for clinical head and neck treatment plans and modifications of said plans with purposely introduced errors. Sensitivity and specificity were compared to the alternative measurement‐based approach using the ArcCHECK phantom. The aim of this analysis was to determine whether RadCalc reliably detects the introduced errors and to derive decision thresholds.

## 2 METHODS

### 2.1 Treatment plans

**FIGURE 1:**
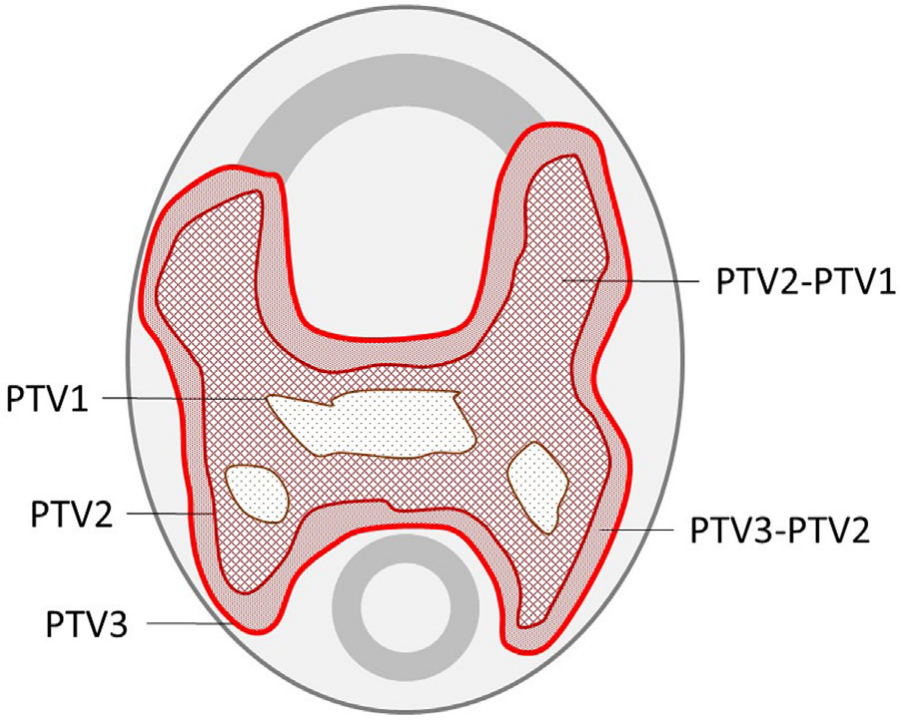
Sketch of the target volumes. PTV1 is the smallest volume receiving the highest prescription dose and part of PTV2; PTV2is part of PTV3. Doses are prescribed to PTV shells, such as PTV2‐PTV1, which is the PTV2 volume excluding the PTV1 volume.

Thirty clinical treatments plans were retrospectively extracted from the institution database. All met the following criteria: Irradiation of the head and neck region with an integrated boost to three dose levels, planned with Pinnacle (Philips Radiation Oncology Systems, Milpitas, California, USA, version 16.2) treatment planning system (TPS) using the Autoplanning module and intended for an Elekta Synergy linear accelerator with an Agility multileaf collimator (Elekta Oncology Systems, Crawley, UK) at 6 MV. Half the plans had three prescribed dose levels of 66 Gy/60 Gy/54 Gy delivered in 30 fractions. The others had 69.3 Gy /66.0 Gy/56.1 Gy delivered in 33 fractions. All doses were prescribed to the D95% dose level. The target volumes are referred to as PTV1, PTV2 and PTV3 (Figure 1), with PTV1 receiving the highest prescription dose, PTV2‐PTV1 the medium dose and PTV3‐PTV2 the lowest dose. For dose calculation, the collapsed cone convolution dose engine and a 3 mm × 3 mm × 3 mm dose grid were utilized. The majority of plans consisted of two full VMAT arcs.

In addition to the clinical plans, 30 errors were purposely introduced into 18 of the treatment plans. This was achieved by editing the plan in the TPS in one of the following ways:

- Densities in the planning computed tomography (CT) were incorrectly overridden. Instead of the interior of a given structure, everything except the interior was overridden with the specified density. The structures complementary to the couch (three cases) with a density of 0.6 g/cm^3^ or complementary to a metallic structure with the material specific density (four cases) was overridden incorrectly in such a way. Such inverted density overrides are a peculiarity of the TPS Pinnacle used in this study. RadCalc ignores the inversion of such density overrides.
- The area below the couch in the CT images is usually excluded from the dose calculation. Here, the cutoff plane used in the TPS was deliberately shifted in such a way that part of the body density was excluded from the dose calculation (four cases).
- Instead of the 6 MV beam model, the plans were calculated using a 6 MV flattening‐filter free (FFF) beam (four cases) or a 10 MV beam of the same treatment machine (4 cases). These represent rather extreme modelling errors or cases in which commissioning data has been mixed up.
- Modelling errors in the beam configuration of the TPS were introduced by modifying the commissioned machine parameters. This was performed in such a way that the newly resulting profiles (six cases) or output factors (five cases) deviated from the previously commissioned data by defined amounts, corresponding to typical differences when using various uncorrected small field detector measurements. These represent, for the most part, subtle modelling errors. The model variants correspond to models 4, 5, 7, 8, 9, 10, 11, and 13 described elsewhere.^21^

A plan was defined as erroneous if any of the DVH parameters listed in Table 1 deviated by at least 2% from the original clinical plan. Generally, prescribed doses should be accurate to within 3–5% over all the possible uncertainties, of which the dose calculation is just one aspect.^22^, ^23^, ^24^ Therefore, the slightly lower threshold of 2% was chosen for the contribution of the dose calculation, which also agrees with the clinically acceptable variation of the prescribed dose versus dose achieved during treatment plan optimization. Whenever this procedure of introducing errors led to an erroneous plan, the plan was included for further analysis. The number of plans corresponding to each error type is given in parentheses in the above list of error types. The listed criteria are routinely used in our institution to assess plan quality. All relevant organs at risk (OAR) were contoured for the plans. As they varied between the different cases due to the tumor location, only those commonly available in almost all studied plans were included, namely, the spinal canal and brainstem (contoured in all cases), and the parotid glands (left parotid not contoured in two cases, right parotid not contoured in one case).

**TABLE 1:** List of DVH parameters

Structure	DVH parameter
PTV1	D_95%_
PTV1	D_50%_
PTV1	D_98%_
PTV1	D_02%_
PTV2‐PTV1	D_95%_
PTV2‐PTV1	D_50%_
PTV2‐PTV1	D_98%_
PTV3‐PTV2	D_95%_
PTV3‐PTV2	D_50%_
PTV3‐PTV2	D_98%_
PTV3‐(PTV2+0.5)	D_02%_
Spinal canal	D_1ccm_
Brainstem	D_1ccm_
Left parotid	D_66%_
Right parotid	D_66%_

Any deviation of more than 2% in any of these parameters in the calculation of modified plans in the TPS compared to the original clinical plan classified the modified plan as erroneous.

For validation, seven head and neck clinical treatment plans fitting the above‐mentioned fractionation scheme newly created since the extraction of the 30 plans for the initial evaluation were analyzed. For each of these plans one error was implemented. Errors included one plan with an inverted density override of the couch structure, one plan in which the couch and couch removal were positioned within the patient contour, one plan including an energy change from 6 to 10 MV, one in which the flattening filter was removed and the usage of three incorrect beam models.

Data (plan, dose distribution, image data, and structure sets) of all clinical treatment plans and all modified error plans were exported in DICOM format for subsequent import into the secondary dose calculation software.

### 2.2 Secondary dose calculation

Secondary dose calculation was performed with RadCalc (LAP Laser GmbH, Lueneburg, Germany, version 7.2). The beam model in RadCalc was created by the vendor. The model was based on measured beam data we provided, which should ideally be data collected independently from the data used for TPS commissioning. This way, secondary dose calculation is able to identify discrepancies in dose calculation, for example, due to incorrect beam modelling or incorrectly entered or labelled beam data. RadCalc requires image data from the TPS. HU to density is converted in RadCalc using tabular data that has to be entered for each scanner. The software obtains the PTV and OAR contours from the structure set including the allocated density overrides. In certain planning systems, density inversions are possible, such as overriding everything outside of a particular region of interest with a specified density. Such inversions of overrides are ignored by RadCalc. Density specified for a structure is always attributed to the inside of that structure. RadCalc has different ways of dealing with lower density elements outside of the patient. Here, the outline and all support structures were used for dose calculation rather than the whole CT. Dose calculation cutoff lines specified in the TPS are ignored.

In order to use a different algorithm type than in the TPS, the analysis was performed using the Monte Carlo dose calculation option. The uncertainty was specified as 1% within 50% of the maximum dose. The dose grid size was set to 2 mm × 2 mm × 2 mm. All plans were recalculated and then compared to the TPS calculation using various metrics. Gamma analysis was performed using global, absolute gamma and a 10% low dose threshold, applying the criteria 5%/3 mm, 3%/3 mm, 3%/2 mm, 2%/2 mm and 1%/1 mm. Alternatively, the maximum dose deviation (max. dev.) of all the DVH parameters of the targets and OARs listed in Table 1 between the secondary dose calculation and the initial TPS dose calculation of each plan was determined. Analysis of DVH parameters of interest was easily configurable by using evaluation templates.

### 2.3 Phantom QA

All unmodified and modified treatment plans were copied onto the virtual model of the ArcCHECK phantom (Sun Nuclear Corporation, Melbourne, Florida, USA). This procedure implies that all density overrides used in the patient anatomy have no impact and the cutoff line below the couch is shifted to fit the phantom geometry. Dose distributions on the virtual phantom were computed and then exported for comparison with measurements.

Measurements of all unmodified plans were compared to the calculated dose distributions of the respective unmodified and modified plans using SNCPatient software (Sun Nuclear Corporation, version 6.7.3). Passing rates were determined using an absolute, global gamma test^1^ with a 10% low‐dose threshold at the criteria of 3%/3 mm, 3%/2 mm and 2%/2 mm. Gamma analysis was performed on a plan‐basis, meaning cumulative for all treatment fields of one plan.

### 2.4 ROC curves

ROC curves were generated for the different criteria described for both the secondary dose calculation (Section 2.2) and the phantom QA (Section 2.3). The curves are generated in such a way that the decision threshold values are varied, and the respective sensitivity and 1‐specificity are calculated and plotted. Subsequently, the point on the curve with the largest distance from the diagonal line going through the plot origin needs to be identified. The decision threshold used to generate this data point is set as the optimal decision threshold, providing a good combination of sensitivity and specificity.

## 3 RESULTS

### 3.1 Secondary dose calculation thresholds

Introduced errors were of the following magnitude as judged from comparing two TPS calculations: six of the induced errors did not affect target DVH parameters but only OAR parameters by more than 2%. In nine plans, target DVH parameters deviated between 2% and 5% at maximum. The other 15 plans exceeded 5% change in target DVH parameters. Regarding the OAR dose, only two plans contained a change of OAR DVH parameters less than 2%, three a change between 2% and 5%, and the vast majority had changes greater than 5%. Maximum change in dose parameters of the OARs was 44%. All errors in the seven validation plans led to at least a 2% change in one of the reported DVH parameters of either target volumes or relevant OARs.

**FIGURE 2:**
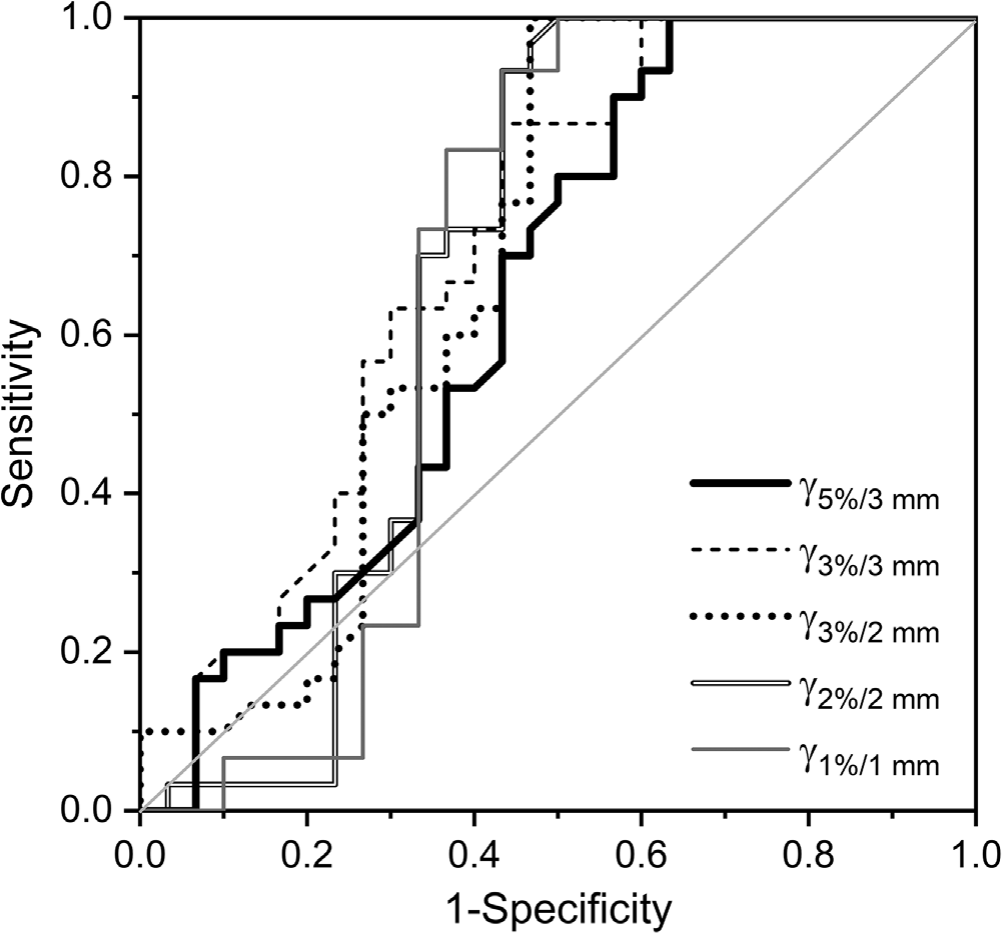
ROC curve obtained for the calculation‐based QA using RadCalc and evaluating various gamma criteria.

Using gamma analysis in evaluating secondary dose calculations, the five studied gamma criteria yielded generally similar ROC curves (Figure 2). AUC values (Table 2) are comparable within the uncertainties. The highest AUC was obtained for the 2%/2 mm criterion and the lowest AUC for the 3%/3 mm criterion. Applying the obtained decision threshold for the 2%/2 mm criterion, 1 out of 30 unmodified treatment plans was falsely flagged as erroneous and 16 of 30 modified treatment plans remained undetected. The latter group of plans included in particular beam modelling errors, including errors in all beam model variants.

**TABLE 2:** AUC and optimum decision threshold from the ROC analysis for the different QA criteria under investigation

	Criterion	AUC	Decision threshold [%]
RadCalc	Gamma 5%/3 mm	0.65 (0.07)	87.3
	Gamma 3%/3 mm	0.71 (0.07)	98.0
	Gamma 3%/2 mm	0.69 (0.07)	93.2
	Gamma 2%/2 mm	0.68 (0.08)	89.6
	Gamma 1%/1 mm	0.67 (0.08)	64.4
	max. dev. target	0.86 (0.05)	1.5
	max. dev. OAR	0.71 (0.08)	12.8
ArcCHECK	Gamma 3%/3 mm	0.78 (0.06)	98.3
	Gamma 3%/2 mm	0.76 (0.06)	96.5
	Gamma 2%/2 mm	0.80 (0.06)	91.1

Numbers in brackets indicate the standard uncertainties.

**FIGURE 3:**
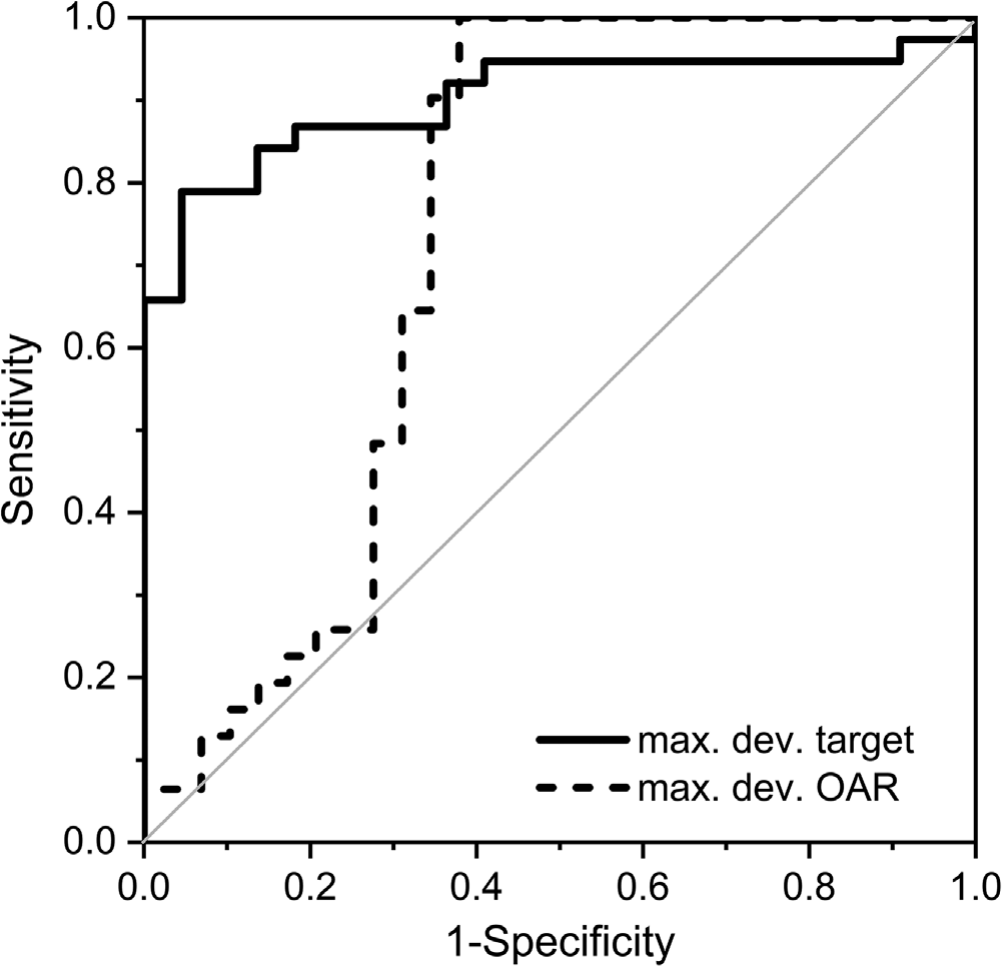
ROC curve obtained for the calculation‐based QA using RadCalc and evaluating the maximum deviation of target and OAR structures.

By means of secondary dose calculation using the max dev. criterion, the evaluation of target structures yielded the highest AUC of all studied criteria (Figure 3, Table 2). The AUC of the max. dev. criterion for the OARs is comparable to the values obtained by secondary dose calculation and gamma analysis. Applying the obtained decision threshold for max. dev. target, nine unmodified treatment plans were falsely marked as erroneous and four modified treatment plans remained undetected. The latter group included only plans created with altered beam models (model variants affecting small field output factors and penumbra).

### 3.2 Phantom QA thresholds

**FIGURE 4:**
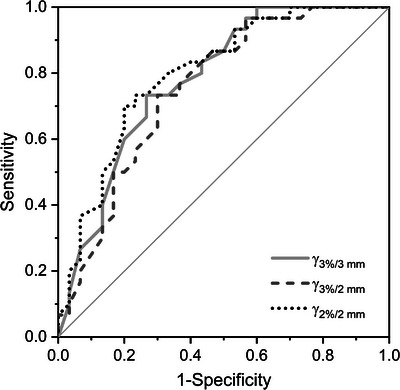
ROC curve obtained for the measurement‐based QA using the ArcCHECK phantom and evaluating various gamma criteria.

For phantom measurements, all three studied gamma criteria yielded similar ROC curves (Figure 4) with comparable AUC (Table 2). The stricter gamma criteria 2%/2 mm yielded slightly higher AUC. Applying this criterion with the obtained decision threshold of 91.1%, nine unmodified treatment plans were falsely marked as erroneous and seven modified treatment plans remained undetected. The latter included especially the density‐related errors and some of the beam modelling errors (model variants affecting small field output factors and penumbra).

### 3.3 Validation results

Applying the obtained thresholds to assess the 14 plans used for validation (seven unmodified, plus seven modified), false positives and false negatives were obtained as detailed in Table 3. Overall, sensitivity was usually high for the three studied criteria, including perfect sensitivity for the RadCalc gamma analysis (Table 3a). Specificity was poorer, especially for the RadCalc gamma analysis (Table 3a) but also for the ArcCHECK gamma analysis (Table 3c). RadCalc in combination with an evaluation of the target DVH parameters had the highest specificity (Table 3b).

**TABLE 3:** Classisfication of the validation plans using the different QA methods employing the decision thresholds according to Table 2: (a) RadCalc in combination with gamma analysis (2%/2 mm), (b) RadCalc in combination with the max. dev. target criterion, (c) ArcCHECK in combination with gamma analysis (2%/2 mm).

(a)		
	Unmodified clinical plan	Modified error plan
QA accepted	7	5^a^
identified as error	0	2

(b)		
	unmodified clinical plan	modified error plan
QA accepted	6	1^b^
identified as error	1	6

(c)		
	unmodified clinical plan	modified error plan
QA accepted	6	3^c^
identified as error	1	4

^a^ undetected errors: couch and couch removal positioned within the patient contour, energy change, 3 incorrect beam models

^b^ undetected error: incorrect beam model

^c^ undetected errors: inverted density override of the couch structure, couch, and couch removal positioned within the patient contour, incorrect beam model

As an example, a plan including an error introduced by a wrong beam model yielded an 88.2% gamma passing rate for the 2%/2 mm criterion on the ArcCHECK phantom, and was thus correctly identified as erroneous. The same plan yielded a 94.3% gamma passing rate for the 2%/2 mm criterion using RadCalc, being above the decision threshold, so the error was not detected. However, DVH‐based analysis using RadCalc did reveal discrepancies of 3% in the target volume parameters and −16.3% in the dose to the right parotid, correctly indicating the plan to contain a calculation error. A second example of an inverted density override was not detectable on ArcCHECK (91.9% gamma passing rate for 2%/2 mm), but yielded an extremely reduced gamma passing rate for RadCalc (35.7% for 2%/2 mm) as well as clear errors up to 46.3% in D98% target volume DVH‐parameter. This illustrates the superior error detection capability of the DVH‐based secondary dose calculation. Alternatively, it also suggests that it can be helpful to combine different QA approaches, that is, secondary dose calculation and phantom QA.

## 4 DISCUSSION

The purpose of the ROC analysis was a comparison of different QA methodologies and the systematic derivation of optimal decision thresholds. Of the three compared approaches, secondary dose calculation in conjunction with gamma analysis provided the smallest area under the ROC curve. Phantom QA on the ArcCHECK and secondary dose calculation with RadCalc based on target DVH parameters resulted in a higher AUC (>0.8). The validation cases confirm the suitability of the proposed decision thresholds. The number of available validation cases was limited due to a different treatment planning system starting to be used for clinical cases and moving most head and neck patients to a different type of linear accelerator.

None of the studied tools provided perfect sensitivity in combination with high specificity. A practical approach may therefore be the combination of different tools. Xu et al. suggested independent dose calculation to be used for screening plans that need to be measured.^15^

The measurement‐based QA approach failed to reliably detect errors related to density irregularities applied to the patient CT and some of the beam modeling errors affecting small field output factors and penumbra. All errors connected to density issues cannot be detected when the plan is copied to the phantom for calculation. Changes in the beam model may go unnoticed due to detector resolution, uncertainties allowed for setup and the nature of gamma evaluation, especially when such changes affect the dose gradient region.

Secondary dose calculation alone does not check whether the plan is correctly deliverable. To complement the secondary dose calculation, RadCalc and other vendors also offer log file analysis, which we did not evaluate under the scope of this project. It is obvious that secondary dose calculation software is unable to detect machine‐specific errors, so such errors were not included in the analysis. Certainly, machine‐specific QA needs to be emphasized when plan‐specific QA is frequently replaced by calculation.

Based on the results, evaluation of the secondary dose calculation using the target DVH parameters (max. dev. target) can be recommended for RadCalc rather than gamma analysis. Directly evaluating DVH parameters is a huge advantage when all relevant structures are included in the evaluation templates, as the clinical meaning of a slightly reduced gamma passing rate is difficult to establish.

The evaluation of the DVH parameters for the OARs shows a much lower AUC. This is due to increasing discrepancies between the secondary dose calculation model and the TPS model further out of field. This needs to be investigated and the models reconsidered. Thereafter, deviation in DVH parameters of the OARs should be considered as an additional criterion for plan‐specific QA. This should also improve the system's sensitivity to beam modelling errors, especially when those are present out of field and, thus, affect the OARs more than the target structures.

Secondary dose calculation in combination with gamma analysis especially failed to detect beam modelling errors of all types and some planning CT density related errors. When used in combination with a DVH‐based analysis beam modelling errors affecting small field output and penumbra failed to be detected.

## 5 CONCLUSION

Secondary dose calculation with RadCalc is an alternative to established measurement‐based phantom QA. Pass/fail decisions based on target DVH criteria yielded the most efficient criterion to detect the purposely implemented errors in patient CT density, beam energy and beam modelling. Due to a secondary dose calculations inherent inability to detect machine errors, a combined approach with measurement‐based QA and intensified machine‐specific QA seems reasonable.

## AUTHOR CONTRIBUTIONS

Sonja Wegener: Conceptualization, Investigation, Interpretation of Data, Writing—Original draft preparation. Ruaa Abu Rashed: Conceptualization, Investigation, Interpretation of Data, Writing—Review and Editing. Otto A. Sauer: Interpretation of Data, Writing—Review and Editing. Gary Razinskas: Conceptualization, Interpretation of Data, Writing—Review and Editing.

## CONFLICT OF INTEREST STATEMENT

The authors declare no conflicts of interest.
